# Anti-IgE Response in Human Airways: Relative Contribution of Inflammatory Mediators

**DOI:** 10.1155/S0962935194000505

**Published:** 1994

**Authors:** I. Gorenne, C. Labat, X. Norel, H. Sosse Alaoui, J.-P. Gascard, C. Brink

**Affiliations:** CNRS URA 1159 Centre Chirurgical Marie-Lannelongue 133, avenue de la Resistance Le Plessis-Robinson 92350 France

## Abstract

Heman airway preparations at resting tone were relaxed with either the leukotriene synthesis inhibitor BAY x1005 (3 μM), chlorpheniramine (1 μM) or the thromboxane receptor antagonist BAY u3405 (0.1 μM). The response to anti-IgE (1:1000) was 58 ± 8% of acetylcholine pre-contraction (2.19 ± 0.28 g). Indomethacin (3 μM) enhanced the anti-IgE-induced contraction by 28%. The anti-IgE maximal response was not modified by either chlorpheniramine, BAY x1005 or BAY u3405. When the tissues were treated with either BAY xl005/indomethacin or BAY x1005/chlorpheniramine, the anti-IgE-induced contraction was reduced. In addition, in presence of BAY xl005/indomethacin/chlorpheniramine the response was completely blocked. These results suggest that mediatots released during anti-IgE challenge cause airway contraction which may mask the evaluation of the leukotriene component.


**Research Paper**

Mediators of Inflammation 3, 359–363 (1994)

### Anti-IgE response in human airways: relative contribution of inflammatory mediators

I. Gorenne, C. Labat, X. Norel, H. Sosse Alaoui, J.-P. Gascard and C. Brink[CA]

CNRS URA 1159, Centre Chirurgical Marie-Lannelongue, 133, avenue de la Resistance, 92350 Le Plessis-Robinson, France

[CA] Corresponding Author

**Human airway preparations at resting tone were relaxed with either the leukotriene synthesis inhibitor BAY x1005 (3 μM), chlorpheniramine (1 μM) or the thromboxane receptor antagonist BAY u3405 (0.1 μM). The response to anti-IgE (1:1000) was 58 ± 8% of acetylcholine pre-contraction (2.19 ± 0.28 g). Indomethacin (3 μM) enhanced the anti-IgE-induced contraction by 28%. The anti-IgE maximal response was not modified by either chlorpheniramine, BAY x1005 or BAY u3405. When the tissues were treated with either BAY x1005/indomethacin or BAY x1005/ chlorpheniramine, the anti-IgE-induced contraction was reduced. In addition, in presence of BAY x1005/indomethacin/chlorpheniramine the response was completely blocked. These results suggest that mediators released during anti-IgE challenge cause airway contraction which may mask the evaluation of the leukotriene component.**

**Key words**: Anti-IgE, BAY x1005, Contraction, Human airways, Indomethacin, Leukotriene synthesis inhibitor

### Introduction

Challenge of airway smooth muscle *in vitro* with an appropriate antigen provokes the release of a variety of inflammatory mediators.[1–3] Data obtained in guinea-pig respiratory tissues demonstrated the release of histamine during the contraction induced by antigen as well as metabolites of both the cyclooxygenase and 5-lipoxygenase pathways.[4–7] Adams and Lichtenstein[8] showed that human airways when passively sensitized and challenged with ragweed antigen, an initial histamine response followed by a leukotriene (LT) component in the contraction could be observed. Undem *et al.*[9] described a potential role for products of the cyclooxygenase pathway, specifically $PGE_2$, in regulating histamine release in human bronchial muscles preparations. In addition, these investigators also demonstrated an increased quantity of LTs in the presence of indomethacin (Ind) when human airways were challenged with antigen. In contrast, other authors have shown inhibitory, or no effects, of Ind on antigenic challenge in human airways.[10–13] Recently, Björck and Dahlén[14] have reported that potent LT antagonists and/or 5-lipoxygenase inhibitors decreased the anti-IgE induced contractions. In other studies, such inhibition could be seen only in presence of additional inhibitors or antagonists.[15–17] This study was undertaken in order to define the relative contribution of each inflammatory mediator implicated in anti-IgE-induced contraction in human isolated bronchial muscle preparations. The experiments were performed using combinations of potent inhibitors and antagonists of inflammatory mediators, including BAY x1005 which is a selective LT synthesis inhibitor in human bronchi.[17]

### Materials and methods

*Experimental protocol*: Human lung tissues were obtained from patients who had undergone thoracotomy for lung carcinoma. Subsequent to the resection of a lung or a lobe, segments of bronchi were dissected free of parenchymal tissue and placed in Tyrode's solution. The preparations were stored at 4°C and used within 18 h. Bronchial ring preparations (2–4 mm of internal diameter) were set up in an organ bath (initial loads of 2–2.5 g) in Tyrode's solution at 37°C aerated with 5% $CO_2$ in $O_2$. The composition of the Tyrode's solution was (millimolar concentrations): NaCl, 139.2; KCl, 2.7; $CaCl_2$, 1.8; $MgCl_2$, 0.49; $NaHCO_3$, 11.9; $NaH_2PO_4$, 0.4 and glucose, 5.5; pH 7.4. Isometric force–displacement transducers (Narco, F-60) and Linseis physiographs were used to record the changes in force. The tissues were allowed to equilibrate for 90 min then contracted with acetylcholine (ACh; 100 μM) and subsequently washed until basal tone was re-established. Bronchial ring preparations were challenged with different dilutions of anti-IgE using either an individual or cumulative dosing method. In another series of protocols, different preparations were exposed for 30 min to either: vehicle (control), the $H_1$ antagonist chlorpheniramine (Chl; 1 μM), the cyclooxygenase inhibitor indomethacin (Ind; 3 μM), the LT synthesis inhibitor BAY x1005 (3 μM) or a combination of these drugs at the same concentrations. Other preparations were treated with the thromboxane (TP)

© 1994 Rapid Communications of Oxford Ltd receptor antagonist BAY u3405 (0.1 μM). At the end of this incubation, the tissues were challenged with anti-IgE (dilution 1:1000). Some untreated preparations were stimulated with anti-IgG (1:1000 of dilution).

*Calculation of results*: The ACh (100 μM) response and the effect of drugs on the basal tone were expressed in g. Relaxation was indicated by a negative symbol (–). Anti-IgE maximal responses are presented as percent of the initial contraction induced by ACh. Results are means ± S.E.M. obtained from (*n*) lung samples. Statistical significance was performed using the Student's *t*-test and were considered to be significant at $p < 0.05$.

*Drugs*: The drugs and their sources were: histamine dihydrochloride, acetylcholine chloride, chlorpheniramine maleate, and indomethacin (Sigma Chemical Company, St. Louis, MO, USA); anti-(human IgE) and anti-(human IgG) (Nordic Immunological Laboratories, Tilburg, The Netherlands). BAY u3405 and BAY x1005 were a gift from Bayer plc UK. These two latter drugs were diluted in DMSO and further dilutions were performed in Tyrode's buffer. The final dilution of DMSO was less than 1/10 000 and did not alter the bronchial resting tone and anti-IgE response.

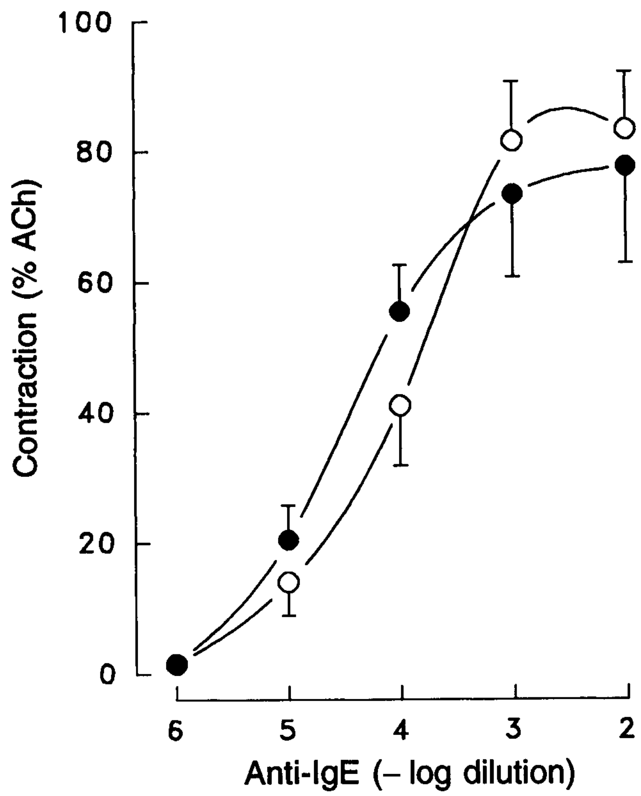

FIG. 1. Anti-IgE-induced contractions in human isolated airways. Bronchial rings were challenged with different dilutions of anti-IgE using either cumulative (●) or non-cumulative (O) method. Data are presented as percent of the acetylcholine (ACh; 100 μM) contractions which were 3.36 ± 0.71 g and 2.44 ± 0.47 g for anti-IgE cumulative or non-cumulative curves, respectively. Values are means ± S.E.M. obtained from five lung samples.

### Results

Human isolated bronchial muscle preparations contracted when anti-IgE was added to the tissues using either a cumulative or individual dosing method (Fig. 1). The results obtained using both methods were not significantly different. Anti-IgG did not produce any contraction of preparations (n = 3). The effects of the different drugs on the resting tone of human airways are presented in Table 1 and Fig. 2. Ind (3 μM) did not significantly alter the resting tone. BAY x1005 (3 μM) or Chl (1 μM) relaxed the preparations either in the presence or absence of Ind. The relaxation induced by the combination of Chl/BAY x1005 was not significantly different from preparations treated only with BAY x1005. BAY u3405 (0.1 μM) also relaxed bronchial muscle basal tone. The contractions induced by anti-IgE (1:1000) were significantly enhanced (28%) following Ind (3 μM) treatment and in tissues treated with the combination Ind/Chl. The anti-IgE-induced contraction after incubation with BAY x1005 was not different from the data obtained following incubation with BAY x1005/Ind. In contrast, the response of Chl treated preparations was significantly reduced compared with the response observed in Ind/Chl treated tissues. The anti-IgE-induced maximal contraction was not modified by either Chl (1 μM), BAY x1005 (3 μM) or BAY u3405 (0.1 μM). In contrast, when BAY x1005 was combined with either Ind or Chl, the anti-IgE-induced contraction was reduced by 37% and 82% when data were compared with either Ind or Chl treated tissues, respectively. In addition, in tissues treated with these three drugs, the anti-IgE responses were completely blocked. In the absence of Ind, the effects of Chl were not different from those of BAY x1005. However, in tissues treated with Ind/Chl the response was markedly increased when compared with contractions obtained in tissues treated with Ind/BAY x1005.

### Discussion

Human bronchial ring preparations contracted in a dose-dependent manner when anti-IgE was added to the tissues, data which support previous results.[18] An endogenous release of mediators may be involved in the basal tone of human airways *in vitro*, since relaxations were observed in tissues treated with Chl, BAY x1005 or BAY u3405. The results are consistent with the relaxations of human airway tone observed in previous reports.[17,19,20] In tissues treated with either the TP receptor antagonist BAY u3405, which blocks the effects of contractile prostanoids,[19] or the antihistamine Chl, there was no alteration in anti-IgE-in-

**Table 1.** Effects of drug treatments on basal tone and anti-IgE response in human airways

| Treatment | No. of lung samples (*n*) | ACh (g) | Basal tone (g) | Anti-IgE (%ACh) |
|---|---|---|---|---|
| Ctrl | 18 | 2.19 ± 0.28 | 0.04 ± 0.04 | 58 ± 8 |
| BAY x1005 | 6 | 2.01 ± 0.48 | −0.14 ± 0.08 | 38 ± 13 |
| Ind | 18 | 1.80 ± 0.21 | 0.14 ± 0.06 | 81 ± 6 |
| Ind + BAY x1005 | 6 | 1.89 ± 0.44 | −0.12 ± 0.04 | 30 ± 12 |
| Chl | 18 | 2.07 ± 0.28 | −0.23 ± 0.06 | 65 ± 8 |
| Chl + BAY x1005 | 6 | 1.46 ± 0.26 | −0.49 ± 0.18 | 12 ± 4 |
| Ind + Chl | 18 | 1.68 ± 0.28 | −0.24 ± 0.08 | 90 ± 8 |
| Ind + Chl + BAY x1005 | 6 | 2.09 ± 0.46 | −0.41 ± 0.13 | 1 ± 1 |
| BAY u3405 | 8 | 2.31 ± 0.42 | −0.18 ± 0.06* | 63 ± 7 |

Airway preparations were contracted with acetylcholine (ACh; 100 μM), washed until basal tone was re-established and incubated (30 min) with either vehicle (Ctrl), BAY x1005 (3 μM), indomethacin (Ind; 3 μM), chlorpheniramine (Chl; 1 μM), BAY u3405 (0.1 μM) or a combination of these drugs. Effect of drugs on basal tone are presented in g and negative signs indicate relaxation. The maximal response to anti-IgE (1:1000) is expressed as percent of ACh contractions. Values are means ± S.E.M. of *n* lung samples. *Result different from control ($P < 0.05$, Student's *t*-test). All other significances are indicated in Figs 2 and 3.

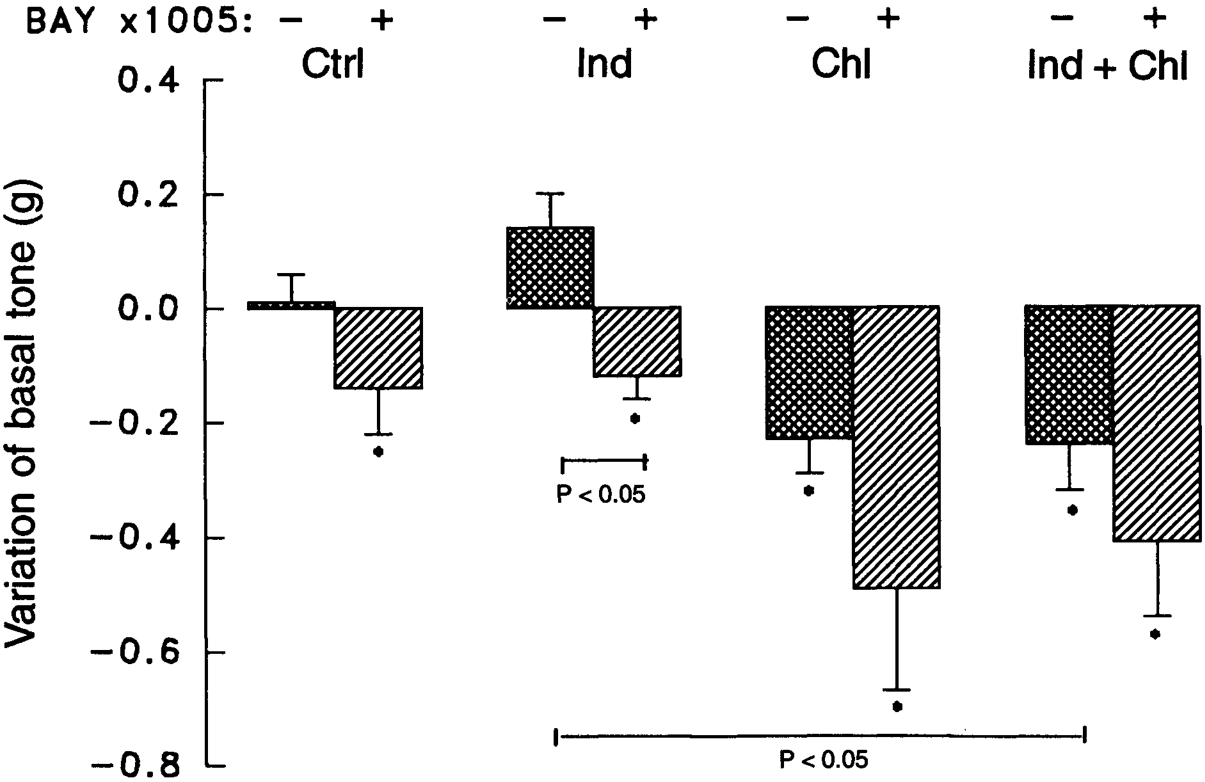

FIG. 2. Effects of drug treatments on human airways resting tone. Tissues were exposed for 30 min to vehicle (Ctrl), BAY x1005 (3 μM), indomethacin (Ind; 3 μM) chlorpheniramine (Chl; 1 μM) or a combination of these drugs at the same concentrations. Responses are expressed in g. Values are means ± S.E.M. *Indicates data significantly different from control (Ctrl) and other values significantly different are shown by horizontal bars ($P < 0.05$, Student's *t*-test).

duced contraction. Ind did not modify resting tone in human airways[21] but the contractile response to antigen was enhanced, results similar to those reported by Adams and Lichtenstein.[22] However, the increase in contraction observed upon antigen challenge of human airways *in vitro* was small (approximately 30%) and in other protocols not observed.[10–13] This slight modulation in human airways is markedly different from that which was reported for guinea-pig tracheal preparations[23] where potentiation of antigen response was considerable (2- to 3-fold) subsequent to the Ind treatment. Undem *et al.*[24] have suggested that in human bronchial muscle preparations prostaglandin $E_2$ may regulate the release of histamine from mast cells during antigen challenge. Therefore, removal of an endogenous inhibitory cyclooxygenase product may explain the enhanced contractile response which may be due to the increased quantity of histamine detected. However, in conditions where only a histamine component remained (Ind/BAY x1005 treated tissues), the response to anti-IgE was not significantly altered when compared with preparations treated with BAY x1005. Therefore, the increased quantity of histamine may

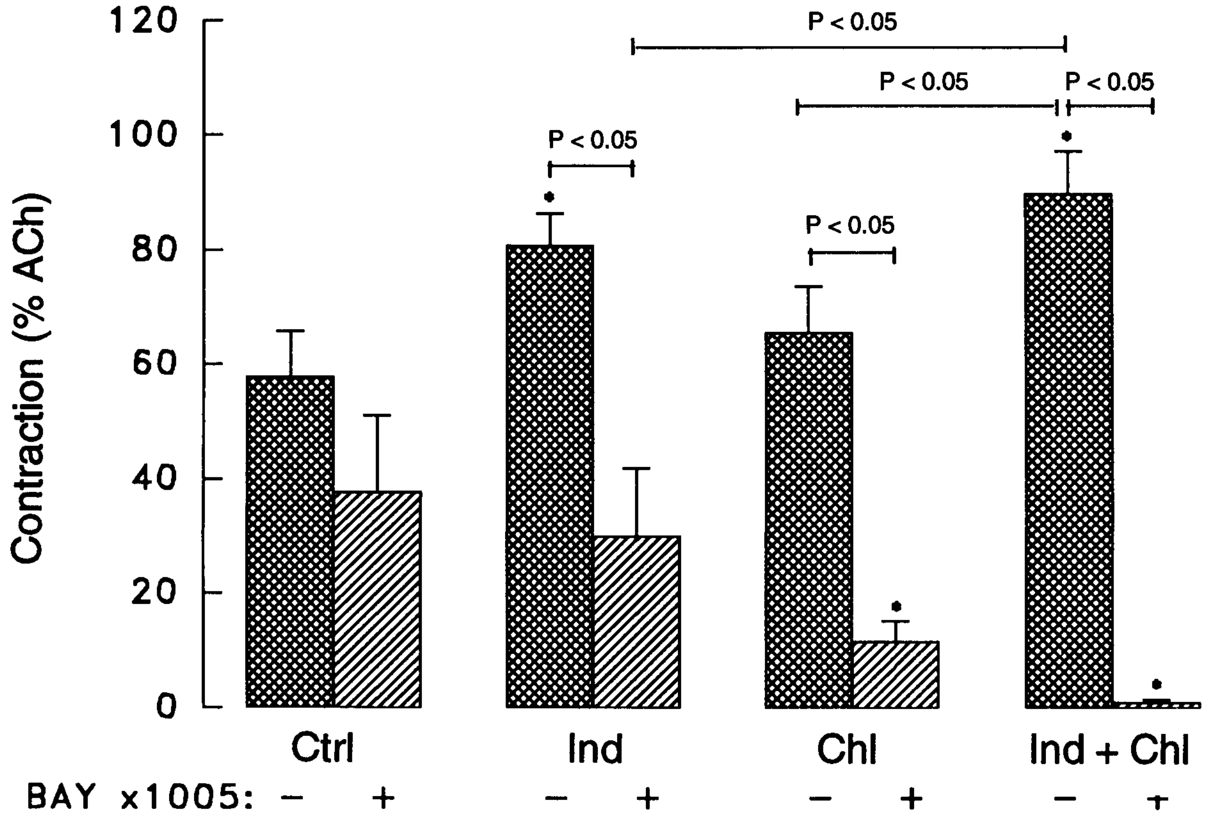

FIG. 3. Effects of drug treatments on anti-IgE-induced contraction of human airways. Tissues were exposed for 30 min to vehicle (Ctrl), BAY x1005 (3 μM), indomethacin (Ind; 3 μM),chlorpheniramine (Chl; 1 μM) or a combination of these drugs at the same concentrations. The tissues were contracted with anti-IgE (1 : 1000). Responses are expressed as percent of acetylcholine (ACh; 100 μM). Values are means ± S.E.M. ACh and the number of lung samples are presented in Table 1. * Indicates data significantly different from control (Ctrl) and other values significantly different are shown by horizontal bars (P < 0.05, Student's *t*-test).

not explain the enhanced contractile response in tissues treated with Ind. In contrast, tissues treated with the combination Ind/Chl where the LT component was present, the contraction was significantly enhanced compared with results obtained in Chl-treated preparations. These latter observations suggest that metabolites of the cyclooxygenase pathway may modulate the LT-induced contraction. A shunting of arachidonic acid to metabolites of the lipoxygenase pathway in the presence of Ind has been suggested by Undem *et al.*[24], where $LTD_4$ and $LTE_4$ were reported to be increased. However, another study[17] did not observe an increased production of $LTE_4$ during anti-IgE stimulation of human airway in the presence of Ind. The data presented in this present report suggest that reduced contraction in the presence of prostaglandins may be due to modulation of LT contraction by metabolites of cyclooxygenase pathway rather than alteration in histamine release. Dahlén *et al.*[18] have recently demonstrated that the contraction provoked by IgE-mediated stimulation of human airways could be antagonized by treatment of the tissues with an LT antagonist (ICI 198,615) or an inhibitor of LT biosynthesis (Piriprost). However, a number of reports using respiratory tissues from different animal species suggested that inhibitors of the 5-LO pathway alone were not effective. Only under specific experimental conditions, namely in the presence of Ind and an antihistamine, were 5-LO inhibitors effective in blocking the antigen induced contraction.[6,16] In addition, Muccitelli *et al.*[7] demonstrated that in human bronchial muscle LT antagonists were more effective in blocking the antigen challenge in human bronchial muscles pretreated with meclofenamate and an antihistamine than in untreated tissues. The present data define the pretreatments necessary to observe the effects of 5-LO inhibitors in human airways challenged with anti-IgE. BAY x1005 has been previously shown to inhibit the $LTE_4$ release induced by anti-IgE on human airways.[17] In the present report, the data show that BAY x1005 did not significantly inhibit the contractions induced by anti-IgE. However, the LT-synthesis inhibitor markedly decreased the contractions induced by anti-IgE when airways were also treated with either Ind, Chl or the combination Ind/Chl. These results indirectly suggest that the other inflammatory mediators which are released during anti-IgE challenge cause a contraction of the bronchial muscle and may mask the evaluation of the LT component. Furthermore, in the absence of prostanoids, the LT contraction is the major component of the anti-IgE contraction.

### References

1. Schild HO, Hawkins DF, Mongar JL, Herxheimer H. Reactions of isolated human asthmatic lung and bronchial tissues to a specific antigen. Histamine release and muscular contraction. *Lancet* 1951; **2:** 376–382.
2. Brocklehurst WE. The release of histamine and formation of a Slow Reacting Substance (SRS-A) during anaphylactic shock. *J. Physiol (Lond)* 1960; **151:** 416–435.
3. Piper PJ, Vane JR. Release of additional factors in anaphylaxis and its antagonism by antiinflammatory drugs. *Nature* 1969; **223:** 29–35.
4. Hand JM, Buckner CK. Effects of selected antagonists on ovalbumin-induced contraction of tracheal strips isolated from the actively sensitized guinea-pig. *Int J Immunopharmacol* 1979; **1:** 189–195.
5. Burka JF, Ali M, McDonald JWD, Paterson NAM. Immunological and non-immunological synthesis and release of prostaglandins and thromboxanes from isolated guinea-pig tracheas. *Prostaglandins* 1981; **22:** 683–691.
6. Hand JM, Schwalm SF, Lewis AJ. Antagonism of antigen-induced contraction of isolated guinea-pig trachea by 5-lipoxygenase inhibitors. *Int Arch Allergy Appl Immunol* 1986; **79:** 8–13.
7. Muccitelli RM, Tucker SS, Hay DWP, Torphy TJ, Wasserman MA. Is the guinea-pig trachea a good *in vitro* model of human large and central airways? Comparison on leukotriene-, methacholine-, histamine-, and antigen-induced contractions. *J Pharmacol Exp Ther* 1987; **243:** 467–473.
8. Adams GK, Lichtenstein LM. Antagonism of antigen-induced contraction of guinea pig and human airways. *Nature* 1977; **270:** 255–256.
9. Undem BJ, Pickett WC, Lichtenstein LM, Adams GK. The effects of indomethacin on immunologic release of histamine and sulfidopeptide leukotrienes from human bronchus and lung parenchyma. *Am Rev Respir Dis* 1987; **136:** 1183–1187.
10. Dunlop LS, Smith AP. Reduction of antigen-induced contraction of sensitized human bronchus *in vitro* by indomethacin. *Br J Pharmacol* 1975; **54:** 495–497.
11. Dahlén S-E, Hansson G, Hedqvist P, Björck T, Granström E, Dahlén B. Allergen challenge of lung tissue from asthmatics elicits bronchial contraction that correlates with the release of leukotrienes $C_4$, $D_4$ and $E_4$. *Proc Natl Acad Sci* 1983; **80:** 1712–1716.
12. Creese BR, Temple DM. The mediators of allergic contraction of human airway smooth muscle: a comparison of bronchial and lung parenchymal strip preparations. *Clin Exp Pharmacol Physiol* 1986; **13:** 103–111.
13. Norel X, Haye-Legrand I, Labat C, Benveniste J, Brink C. Antigen-induced contraction of human isolated lung preparations passively sensitized with monoclonal IgE: effects of indomethacin. *Int Arch Allergy Appl Immunol* 1988; **87:** 342–348.
14. Björck T, Dahlén S-E. Leukotrienes and histamine mediate IgE-dependent contractions of human bronchi: pharmacological evidence obtained with tissues from asthmatic and non-asthmatic subjects. *Pulmonary Pharmacology* 1993; **6:** 87–96.
15. Jones TR, Charette L, Denis D. Leukotriene and anti-IgE induced contractions of human isolated trachea: studies with leukotriene receptor antagonists and a novel 5-lipoxygenase inhibitor. *Can J Physiol Pharmacol* 1988; **66:** 762–768.
16. Cho HL, Ho PPK, Mihelich ED, Snyder DW. Relative potencies of 5-lipoxygenase inhibitors on antigen induced contractions of guinea-pig tracheal strips. *J Pharmacol Meth* 1991; **26:** 277–287.
17. Gorenne I, Labat C, Gascard JP, *et al.* (*R*)-2-[4-(Quinolin-2-yl-methoxy)phenyl-2-cyclopenty] acetic acid (BAY x1005), a potent leukotriene synthesis inhibitor: effects on Anti-IgE challenge in human airways. *J Pharmacol Exp Ther* 1994; **268:** 868–872.
18. Dahlén S-E, Björck T, Kumlin M, Granström E, Hedqvist P. Studies of leukotrienes as mediators in the human lung *in vitro*. *Adv Prostaglandin Thromboxane Leukotriene Res* 1991; **20:** 193–200.
19. Norel X, Labat C, Gardiner PJ, Brink C. Inhibitory effects of BAY u3405 on prostanoid-induced contractions in human isolated bronchial and pulmonary arterial muscle preparations. *Br J Pharmacol* 1991; **104:** 591–595.
20. Ellis JL, Undem BJ. Role of cysteinyl-leukotrienes and histamine in mediating intrinsec tone in isolated bronchi. *Am J Respir Crit Care Med* 1994; **149:** 118–122.
21. Brink C, Grimaud C, Guillot C, Orehek J. The interaction between indomethacin and contractile agents on human isolated airway muscle. *Br J Pharmacol* 1980; **69:** 383–388.
22. Adams GK, Lichtenstein LM. Indomethacin enhances response of human bronchus to antigen. *Am Rev Respir Dis* 1985; **131:** 8–10.
23. Saad MH, Wilson MA, Burka JF. Release of leukotriene $C_4$ from guinea-pig trachea. *Prostaglandins* 1983; **25:** 741–751.
24. Undem BJ, Pickett WC, Lichtenstein LM, Adams GK. The effect of indomethacin on immunological release of histamine and sulfidopeptide leukotrienes from human bronchus. *Am Rev Respir Dis* 1987; **136:** 1183–1187.

**Received 29 April 1994; accepted 25 May 1994**
